# 115 The Association between Burn Characteristics and Body Image in School-Aged Children years 5 to 12

**DOI:** 10.1093/jbcr/irad045.088

**Published:** 2023-05-15

**Authors:** Khushbu Patel, Kate Surette, Pengsheng Ni, Petra Warner, Frederick Stoddard, Lewis Kazis, Jeffrey Schneider, Colleen Ryan, Madeleine McGwin, Ludwik Branski, Tina Palmieri

**Affiliations:** Shriners Children's Boston, Massachusetts General Hospital, Boston, Massachusetts; Shriners Children's - Boston, Boston, Massachusetts; Boston University School of Public Health, Boston, Massachusetts; Shriners Children's Ohio, dayton, Ohio; Harvard Medical School at the Massachusetts General Hospital, Boston, Massachusetts; Boston University School of Public Health, Rehabilitation Outcomes Center at Spaulding, Boston, Massachusetts; Spaulding Rehabilitation Hospital/Harvard Medical School/Rehabilitation Outcomes Center at Spaulding, Boston, Massachusetts; Shriners Children's-Boston, Massachusetts General Hospital, Harvard Medical School, Boston, Massachusetts; Shriners Children's- Boston, Boston, MA, Boston, Massachusetts; Shriners Children's- Texas, University of Texas Medical Branch, Galveston, Texas; UC Davis and Shriners Children's Northern California, Sacramento, California; Shriners Children's Boston, Massachusetts General Hospital, Boston, Massachusetts; Shriners Children's - Boston, Boston, Massachusetts; Boston University School of Public Health, Boston, Massachusetts; Shriners Children's Ohio, dayton, Ohio; Harvard Medical School at the Massachusetts General Hospital, Boston, Massachusetts; Boston University School of Public Health, Rehabilitation Outcomes Center at Spaulding, Boston, Massachusetts; Spaulding Rehabilitation Hospital/Harvard Medical School/Rehabilitation Outcomes Center at Spaulding, Boston, Massachusetts; Shriners Children's-Boston, Massachusetts General Hospital, Harvard Medical School, Boston, Massachusetts; Shriners Children's- Boston, Boston, MA, Boston, Massachusetts; Shriners Children's- Texas, University of Texas Medical Branch, Galveston, Texas; UC Davis and Shriners Children's Northern California, Sacramento, California; Shriners Children's Boston, Massachusetts General Hospital, Boston, Massachusetts; Shriners Children's - Boston, Boston, Massachusetts; Boston University School of Public Health, Boston, Massachusetts; Shriners Children's Ohio, dayton, Ohio; Harvard Medical School at the Massachusetts General Hospital, Boston, Massachusetts; Boston University School of Public Health, Rehabilitation Outcomes Center at Spaulding, Boston, Massachusetts; Spaulding Rehabilitation Hospital/Harvard Medical School/Rehabilitation Outcomes Center at Spaulding, Boston, Massachusetts; Shriners Children's-Boston, Massachusetts General Hospital, Harvard Medical School, Boston, Massachusetts; Shriners Children's- Boston, Boston, MA, Boston, Massachusetts; Shriners Children's- Texas, University of Texas Medical Branch, Galveston, Texas; UC Davis and Shriners Children's Northern California, Sacramento, California; Shriners Children's Boston, Massachusetts General Hospital, Boston, Massachusetts; Shriners Children's - Boston, Boston, Massachusetts; Boston University School of Public Health, Boston, Massachusetts; Shriners Children's Ohio, dayton, Ohio; Harvard Medical School at the Massachusetts General Hospital, Boston, Massachusetts; Boston University School of Public Health, Rehabilitation Outcomes Center at Spaulding, Boston, Massachusetts; Spaulding Rehabilitation Hospital/Harvard Medical School/Rehabilitation Outcomes Center at Spaulding, Boston, Massachusetts; Shriners Children's-Boston, Massachusetts General Hospital, Harvard Medical School, Boston, Massachusetts; Shriners Children's- Boston, Boston, MA, Boston, Massachusetts; Shriners Children's- Texas, University of Texas Medical Branch, Galveston, Texas; UC Davis and Shriners Children's Northern California, Sacramento, California; Shriners Children's Boston, Massachusetts General Hospital, Boston, Massachusetts; Shriners Children's - Boston, Boston, Massachusetts; Boston University School of Public Health, Boston, Massachusetts; Shriners Children's Ohio, dayton, Ohio; Harvard Medical School at the Massachusetts General Hospital, Boston, Massachusetts; Boston University School of Public Health, Rehabilitation Outcomes Center at Spaulding, Boston, Massachusetts; Spaulding Rehabilitation Hospital/Harvard Medical School/Rehabilitation Outcomes Center at Spaulding, Boston, Massachusetts; Shriners Children's-Boston, Massachusetts General Hospital, Harvard Medical School, Boston, Massachusetts; Shriners Children's- Boston, Boston, MA, Boston, Massachusetts; Shriners Children's- Texas, University of Texas Medical Branch, Galveston, Texas; UC Davis and Shriners Children's Northern California, Sacramento, California; Shriners Children's Boston, Massachusetts General Hospital, Boston, Massachusetts; Shriners Children's - Boston, Boston, Massachusetts; Boston University School of Public Health, Boston, Massachusetts; Shriners Children's Ohio, dayton, Ohio; Harvard Medical School at the Massachusetts General Hospital, Boston, Massachusetts; Boston University School of Public Health, Rehabilitation Outcomes Center at Spaulding, Boston, Massachusetts; Spaulding Rehabilitation Hospital/Harvard Medical School/Rehabilitation Outcomes Center at Spaulding, Boston, Massachusetts; Shriners Children's-Boston, Massachusetts General Hospital, Harvard Medical School, Boston, Massachusetts; Shriners Children's- Boston, Boston, MA, Boston, Massachusetts; Shriners Children's- Texas, University of Texas Medical Branch, Galveston, Texas; UC Davis and Shriners Children's Northern California, Sacramento, California; Shriners Children's Boston, Massachusetts General Hospital, Boston, Massachusetts; Shriners Children's - Boston, Boston, Massachusetts; Boston University School of Public Health, Boston, Massachusetts; Shriners Children's Ohio, dayton, Ohio; Harvard Medical School at the Massachusetts General Hospital, Boston, Massachusetts; Boston University School of Public Health, Rehabilitation Outcomes Center at Spaulding, Boston, Massachusetts; Spaulding Rehabilitation Hospital/Harvard Medical School/Rehabilitation Outcomes Center at Spaulding, Boston, Massachusetts; Shriners Children's-Boston, Massachusetts General Hospital, Harvard Medical School, Boston, Massachusetts; Shriners Children's- Boston, Boston, MA, Boston, Massachusetts; Shriners Children's- Texas, University of Texas Medical Branch, Galveston, Texas; UC Davis and Shriners Children's Northern California, Sacramento, California; Shriners Children's Boston, Massachusetts General Hospital, Boston, Massachusetts; Shriners Children's - Boston, Boston, Massachusetts; Boston University School of Public Health, Boston, Massachusetts; Shriners Children's Ohio, dayton, Ohio; Harvard Medical School at the Massachusetts General Hospital, Boston, Massachusetts; Boston University School of Public Health, Rehabilitation Outcomes Center at Spaulding, Boston, Massachusetts; Spaulding Rehabilitation Hospital/Harvard Medical School/Rehabilitation Outcomes Center at Spaulding, Boston, Massachusetts; Shriners Children's-Boston, Massachusetts General Hospital, Harvard Medical School, Boston, Massachusetts; Shriners Children's- Boston, Boston, MA, Boston, Massachusetts; Shriners Children's- Texas, University of Texas Medical Branch, Galveston, Texas; UC Davis and Shriners Children's Northern California, Sacramento, California; Shriners Children's Boston, Massachusetts General Hospital, Boston, Massachusetts; Shriners Children's - Boston, Boston, Massachusetts; Boston University School of Public Health, Boston, Massachusetts; Shriners Children's Ohio, dayton, Ohio; Harvard Medical School at the Massachusetts General Hospital, Boston, Massachusetts; Boston University School of Public Health, Rehabilitation Outcomes Center at Spaulding, Boston, Massachusetts; Spaulding Rehabilitation Hospital/Harvard Medical School/Rehabilitation Outcomes Center at Spaulding, Boston, Massachusetts; Shriners Children's-Boston, Massachusetts General Hospital, Harvard Medical School, Boston, Massachusetts; Shriners Children's- Boston, Boston, MA, Boston, Massachusetts; Shriners Children's- Texas, University of Texas Medical Branch, Galveston, Texas; UC Davis and Shriners Children's Northern California, Sacramento, California; Shriners Children's Boston, Massachusetts General Hospital, Boston, Massachusetts; Shriners Children's - Boston, Boston, Massachusetts; Boston University School of Public Health, Boston, Massachusetts; Shriners Children's Ohio, dayton, Ohio; Harvard Medical School at the Massachusetts General Hospital, Boston, Massachusetts; Boston University School of Public Health, Rehabilitation Outcomes Center at Spaulding, Boston, Massachusetts; Spaulding Rehabilitation Hospital/Harvard Medical School/Rehabilitation Outcomes Center at Spaulding, Boston, Massachusetts; Shriners Children's-Boston, Massachusetts General Hospital, Harvard Medical School, Boston, Massachusetts; Shriners Children's- Boston, Boston, MA, Boston, Massachusetts; Shriners Children's- Texas, University of Texas Medical Branch, Galveston, Texas; UC Davis and Shriners Children's Northern California, Sacramento, California; Shriners Children's Boston, Massachusetts General Hospital, Boston, Massachusetts; Shriners Children's - Boston, Boston, Massachusetts; Boston University School of Public Health, Boston, Massachusetts; Shriners Children's Ohio, dayton, Ohio; Harvard Medical School at the Massachusetts General Hospital, Boston, Massachusetts; Boston University School of Public Health, Rehabilitation Outcomes Center at Spaulding, Boston, Massachusetts; Spaulding Rehabilitation Hospital/Harvard Medical School/Rehabilitation Outcomes Center at Spaulding, Boston, Massachusetts; Shriners Children's-Boston, Massachusetts General Hospital, Harvard Medical School, Boston, Massachusetts; Shriners Children's- Boston, Boston, MA, Boston, Massachusetts; Shriners Children's- Texas, University of Texas Medical Branch, Galveston, Texas; UC Davis and Shriners Children's Northern California, Sacramento, California

## Abstract

**Introduction:**

Children ages 5 to 12 are in pivotal stages of psychological development as they become more self-aware. Body image can be a formation of both internal experiences and influences from the surrounding environment. This perceived image adapts during this age group and can be negatively impacted by the changes in physical appearance due to the burn injury. Previous research indicates older age and the presence of larger, visible burns can impact body image and self-esteem in the present and long-term. This study assessed the association between burn characteristics and the child’s ability to maintain positive body image in this age group using a new parent-reported instrument for assessment of child outcomes after burn injury.

**Methods:**

Preliminary data was obtained from the School-Aged Life Impact Burn Recovery Evaluation (LIBRE)_5-12_ study, which is currently in stages of field-testing. Classic test theory methods were used to assess body image items (n = 13) from the Psychological domain. Each item was scored on a 5-point Likert scale ranging from 0 (never) to 4 (always). Data was recoded for selected items such that higher scores denote better functioning. Multivariate linear regression analyses measured the association between burn injury characteristics and body image (controlling for sex, race and ethnicity, burn injury to critical areas, total body surface area burned (TBSA), and age at survey completion). Mean body image score was calculated.

**Results:**

Data was obtained from 356 surveys with completed body image items from parents of children with burns. The mean age at survey completion was 8.6 + 2.4 years and mean total body surface area burned (TBSA) was 9.2 + 13.9 with 55% male and 66% white. Items from body image subdomain were identified as a unidimensional scale (α = 0.91, item-total correlations >0.4, ratio of the 1st and 2nd eigenvalues = 4.98). The mean body image score was 3.5 + 0.6. The presence of a foot burn (*p* = 0.0099), genitalia burn (*p* = 0.0449) and TBSA >1% (*p* = 0.0013) resulted in a lower body image score (3.3 + 0.6, 3.1 + 0.8, and 3.4 + 0.7, respectively). In the regression model, age at survey completion was also associated with decreased body image score (β = 0.074, *p* < 0.0001).

**Conclusions:**

Parents reported older school-aged burn survivors often had difficulty with body image. Presence of larger burn size and injury to critical areas also worsened body image.

**Applicability of Research to Practice:**

Burn-care providers and family members can utilize body image score to understand the burn survivor’s current body image satisfaction and assist in fostering positive body image after burn injury through assessment and early intervention.

